# Ackee (*Blighia sapida* K.D. Koenig) Leaves and Arils Methanolic Extracts Ameliorate CdCl_2_-Induced Oxidative Stress Biomarkers in *Drosophila melanogaster*

**DOI:** 10.1155/2022/3235031

**Published:** 2022-11-14

**Authors:** Omodele Ibraheem, Tosin A. Oyewole, Adeola Adedara, Amos O. Abolaji, Oluwatobiloba M. Ogundipe, Jude Akinyelu, Chukwuebuka T. Eze, Sarah Albogami, Saqer S. Alotaibi, Oluyomi S. Adeyemi, Gaber El-Saber Batiha, Mohammed Alorabi, Michel De Waard

**Affiliations:** ^1^Plants for Biotechnological Resources Research Group, Department of Biochemistry, Federal University Oye-Ekiti, PMB 373, Oye, Ekiti, Nigeria; ^2^Drug Metabolism and Molecular Toxicology Research Laboratories, Department of Biochemistry, Faculty of Basic Medical Sciences, College of Medicine, University of Ibadan, Oyo, Nigeria; ^3^Nanobiochemistry Research Group, Department of Biochemistry, Federal University Oye-Ekiti, PMB 373, Oye, Ekiti, Nigeria; ^4^Environmental Toxicology Research Group, Department of Biochemistry, Federal University Oye-Ekiti, PMB 373, Oye, Ekiti, Nigeria; ^5^Department of Biotechnology, College of Science, Taif University, P.O. Box 11099, Taif 21944, Saudi Arabia; ^6^Department of Biochemistry, Medicinal Biochemistry, Nanomedicine & Toxicology Laboratory, Landmark University, PMB, Omu-Aran, 1001, Nigeria; ^7^Department of Pharmacology and Therapeutics, Faculty of Veterinary Medicine, Damanhour University, Damanhour 22511, El Beheira, Egypt; ^8^Smartox Biotechnology, 6 rue des Platanes, 38120 Saint-Egreve, France; ^9^L'Institute du thorax, Inserm, Cnrs, Univ Nantes, F-44007 Nantes, France; ^10^Universite de Nice Sophia-Antipolis, LabEx Ion Channels, Science and Therapeutics, F-06560, Valbonne, France

## Abstract

Different ethnomedical benefits have been documented on different parts of Ackee (*Blighia sapida*); however, their roles in ameliorating oxidative damages are not well established. CdCl_2_ inhibitory effects on some oxidative-stress biomarkers and ameliorative potentials of Ackee leaves (AL) and arils (AS) methanolic extracts were studied using *Drosophila melanogaster* as a model. One to 3-day-old *D. melanogaster* flies were orally exposed to different concentrations of CdCl_2_ in their diet for 7 days. The fly's survival profile and negative geotaxis assays were subsequently analysed. Methanolic extracts of AL and AS treatments showed negative geotaxis behaviour, and extracts were able to ameliorate the effect of Cd^2+^ on catalase and GST activities and increase total thiol and GSH levels, while it reduced the H_2_O_2_ generation (*p* ≤ 0.05) when compared to the control. Furthermore, Cd^2+^ exhibited noncompetitive and uncompetitive enzyme inhibition on catalase and GST activities, respectively, which may have resulted in the formation of Enzyme-substrate-Cd^2+^ transition complexes, thus inhibiting the conversion of substrate to product. This study, thus, suggests that the Cd^2+^ mechanism of toxicity was associated with oxidative damage, as evidenced by the alteration in the oxidative stress-antioxidant imbalance, and that the AL and AS extracts possess essential phytochemicals that could alleviate possibly deleterious oxidative damage effects of environmental pollutants such as CdCl_2_. Thus, Ackee plant parts possess essential phytonutrients which could serve as valuable resources in heavy metal toxicity management.

## 1. Introduction

Environmental pollutants are one of the major causes of concern today due to the biohazardous nature of heavy metals in them [1]. Metallic elements such as arsenic, mercury, lead, cadmium, and iron contaminate natural environment by anthropogenic and natural means [2]. These include weathering, soil erosion, industrial discharge, mining, sewage effluents to water body, pesticides contaminating foods, soil, water, and atmosphere which humans are exposed to [3].

Cadmium is one of such environmental toxicants found naturally in ores with zinc, copper, and lead [4]. Natural activities are increasing its environmental levels, which include weathering of cadmium containing rock into soil and erosion into water bodies [5]. Furthermore, humans are daily exposed to cadmium containing materials especially in different industrial and mining sites where they are utilized and processed to usable products [6]. Industrial processes are increasing cadmium usage and activities such as mining, neutron absorber in nuclear plant, and electrode material in nickel-cadmium batteries (due to its superiority and reliability when compared with other similar materials). Although its usefulness as a protective pigment in plastic, glass, and paint industry cannot be overemphasized, yet it has always been a toxicant of concern [7]. Also, cadmium contaminated soil through the use of phosphate fertilizer with high cadmium percentage is one of the foremost ways of cadmium exposure along human food chain. The global range of cadmium content in phosphate fertilizers is between 0.8 and 47 mg/kg/product, while exposure levels of 30–50 *μ*g per day have been reportedly linked to increased risk of bone fracture, cancer, kidney dysfunction, and hypertension in adults [8]. Studies have shown that cadmium is a toxicant found naturally in tobacco leaves, and this increases the risk of certain carcinogenic diseases [9]. Cadmium has been reported to exert its genotoxicity through the production of reactive oxygen species (ROS), inhibiting cell proliferation and inhibiting DNA replication [10]. Reports have shown that different industrial chemicals, including cadmium chloride (CdCl_2_), could cause oxidative stress in various cells and organs of the body such as the bone, lungs, stomach, brain, kidney, blood, liver, and ovaries to name a few [11]. Similarly, reactive nitrogen species (RNS) cause nitrosative stress which has been associated with some diseased conditions [12]. Antioxidants which are commonly derived from natural products may offer protection against oxidative and nitrosative stress [12–14].

*Drosophila melanogaster* is a model organism of choice because of their tiny size (2–3 mm), short reproduction time, the simple and economical way to culture them in the laboratory, and the numerous options for detecting abnormal phenotypes. Furthermore, laudable features such as its high reproductive rate and cheap maintenance when compared to other vertebrate and invertebrate models owing to the fact that it raises few ethical questions have highlighted it as one of the leading invertebrate models for biomedical research and education [15, 16]. For more than a century, the fruit fly has been used to unravel major biological questions. The European Centre for the Validation of Alternative Methods (ECVAM) has therefore recommended it as an alternative research model to vertebrate model because it raises minor ethical concerns [17]. It is now a norm to change one or two constituents commonly used in *D. melanogaster* diets or adding toxicants to be investigated to the diets so as to ascertain the roles of the alterations in various behavioural or developmental paradigms [18]. Measures of lifespan and reproductive rates are also important indicators of these paradigms [19]. Numerous behavioural assays have been designed in such a way that some studies and testing can start as early as the embryo stage while other assays can also be designed to study adult behaviour such as geotaxis [20], learning and memory [21], vision, and taste [22].

Ackee (*Blighia sapida* K.D. Koenig) is a shady tree crop common in Jamaica and parts of West Africa [23, 24]. The fleshy and edible part of the ripened fruit is the arils, and the fleshy arils are used in the preparation of sauce and soup among some ethnic groups in Jamaica and parts of West Africa [25]. In addition, the roots, leaves, capsules, and seeds are known in the folkoric management of several diseases in Benin City, Nigeria [23]. There have been tradomedical claims of Ackee in healing different diseases such as malaria, internal hemorrhage, dysentery, yellow fever, and diabetes since all parts of the tree have been documented to have diverse medicinal properties and have also been used together with other tradomedical materials in West African countries [23, 26].

The antioxidants inherent in these Ackee plant parts are vital to its remarkable therapeutic potential. Antioxidants are bioactive compounds that are present in fruits, vegetables, strawberry, tea, etc. Such bioactive compounds include phenolic compounds, carotenoids, anthocyanin, tocopherol, tannins, and terpenoids. They play significant roles towards the management or treatment of different pathophysiological conditions such as diabetes, high blood pressure, ageing, and numerous others [27].

Reports have shown that some ground water in urban areas has high levels of heavy metals beyond permissible limits [28, 29]. Some of these heavy metals include cadmium, chromium, and arsenic contaminate drinkable water bodies and soil. It has been reported that natural world phenomenon and industrial usage of cadmium containing materials for diverse human uses and benefits constantly increase cadmium environmental levels, which eventually get into human food chain, consequently exposing humans to it [28, 29].

Ackee plants are ubiquitous and well consumed in some localities in West Africa and South America where ground water that is often contaminated with heavy metals is the main source of drinkable water [23, 24]. The biological roles and therapeutic applications which are played by the various Ackee plant parts such as the leaves (AL) and arils (AS), which are mostly consumed, and the stem barks and roots used in traditional remedies towards alleviating or ameliorating cytotoxicity have not been well-reported.

This study was carried out to further provide a well-informed knowledge about the therapeutic potentials of the intrinsic phytochemicals in these Acke plant parts would confer towards the amelioration of CdCl_2_ induced cytotoxicity. This was carried out by elucidating the inhibitory effects of CdCl_2_ on *D. melanogaster* oxidative stress biomarkers and the ameliorative potentials which AL and AS methanolic extracts could confer on behavioural and biochemical disruptions induced by exposure to CdCl_2_.

## 2. Materials and Methods

### 2.1. Chemicals

All chemicals used for the analysis were of high analytical grades. Cadmium chloride was procured from A K Scientific, 30023 Ahern Ave, Union City, CA 94587, United States of America, at a 95% purity; reduced glutathione (GSH) (98% purity), 1-chloro-2,4-dinitrobenzene (CDNB) (99% purity), acetylcholine iodide (99% purity), 5′5′-dithiobis-2-nitrobenzoic acid (DTNB) (98% purity), butylated hydroxytoluene (BHT) (99% purity, dichlorofluorescein diacetate (DCFH-DA) (95% purity), and ethanol (99.2% purity) were purchased from Sigma-Aldrich Chemical Company (St. Louis, MO, USA).

### 2.2. Collection and Preparation of AL and AS

AL and AS were collected and identified from the Idofin area in Oye-Ekiti, Ekiti State, Nigeria (latitude 7°53′21.91^″^N and longitude 5°20′41.35^″^E). Authentication of Ackee plant parts was carried out at the Department of Botany, University of Ibadan, with authentication number UCH-22791. At the Department of Biochemistry Laboratory, Faculty of Science, Federal University Oye-Ekiti, Ekiti State, Nigeria, the plant materials were washed, weighed (AS; 500 g and AL; 400 g), and then blended with 500 ml methanol to give smooth ground paste. Homogenates were left for 5 hours with continuous stirring and were then passed through a fine muslin cloth. The filtrates were then centrifuged at 1,000 rpm for 20 minutes using Thermo Fisher Sorvall Legend Micro 17R, Fresco centrifuge for clarification to remove other insoluble particles. Clarified supernatants were concentrated in a rotary evaporator to 10% initial volume, after which it was evaporated to dryness at 50°C in a hot air oven. The dried methanolic extracts of AL and AS were then stored in an airtight container labelled appropriately.

### 2.3. Collection of Fruit Flies and Treatment

Harwich strain of *D. melanogaster* of both genders (1-3 day old) was cultured, maintained at the Drosophila Laboratory, Department of Biochemistry, University of Ibadan, Nigeria. The flies were reared on cornmeal medium containing 1% *w*/*v* brewer's yeast, 2% *w*/*v* sucrose, 1% *w*/*v* powdered milk, 1% *w*/*v* agar, and 0.08% *w*/*v* nipagin at constant temperature of about 23°C and relative humidity of 60% under 12-hour dark/light cycle conditions until the eggs metamorphosed into young adult fruit flies using the same flies strain.

### 2.4. Phytochemical Analysis of Ackee Methanolic Extracts

High-performance liquid chromatography (HPLC) was used for the phytochemical identification and quantitative analysis of methanolic extracts of AL and AS. The bioactive compounds of alkaloid, flavonoid, saponin, and phenol were analysed.

#### 2.4.1. HPLC System (Alkaloid)

Prior to HPLC analysis, 100 mg each of the extracted samples (AL and AS) were added separately to 10 ml of 70% methanol in a well-labelled borosilicate beaker. The sample mixtures were extracted for 20 minutes in an ultrasonic bath at room temperature. After extraction, the sample mixtures were centrifuged at 10,000 rpm for 10 minutes twice. The supernatant was collected and filtrated through a 0.22 *μ*m membrane filter. The HPLC analysis of methanol extracts of AL and AS was then performed using Agilent 1100 series HPLC system consisting of a quaternary delivery system, an autosampler, and a diode array detector (DAD). The chromatographic analysis was carried out on SHISEIDO UG 120 C18 column. Hamilton microliter syringe (5 *μ*m × 4.6 × 250 mm) was used, and Helium gas was used as a carrier gas at a constant flow rate of 1.0 ml/minute; the mobile phases consist of solvent A (10 mM ammonium acetate contained 0.2% Triethanolamine (TEA) at pH 5.0 adjusted by acetic acid) and solvent B (acetonitrile, ACN). The gradient elution mode was programmed as follows: 27% B for 0-10 minutes, 27-33% B for 10-15 minutes, and 33-95% B for 15-30 minutes. UV detection wavelength was set at 280 nm. The flow rate and injection volume were set at 1 ml/minute and 20 *μ*l, respectively. Ackee extracts were centrifuged with Thermo Fisher Sorvall Legend Micro 17R, Fresco centrifuge at 10,000 rpm for 10 minutes; this was repeated. The standards of different concentrations were prepared for the injection into the HPLC system for calibration and correlation coefficient establishment. The supernatants were collected and filtered through a 0.22 *μ*m membrane filter, and 20 *μ*l aliquots from the filtrate were then injected into the HPLC system following the same procedure as standard mixtures.

#### 2.4.2. HPLC System (Phenol)

The effective removal of the polyphenols was carried out in a two-stage extraction process before carrying out the HPLC analysis.

*(1) Stage 1*. Approximately 50 mg of each sample was extracted with 5 ml of 1 M NaOH for 16 hours on a shaker at ambient temperature [30, 31]. After extraction, the samples were centrifuged at 5000 rpm, rinsed with water, and centrifuged again, and supernatants were combined and placed in a disposable glass tube and heated at 90°C for 2 hours to release the conjugated phenolic compound [32]. The heated extracts were cooled, titrated with 4 M HCL to pH ≤ 2.0, diluted to 10 ml, with deionised water, and centrifuged to remove the precipitate. The supernatant was obtained for subsequent purification, and the residues obtained were extracted further at the second stage.

*(2) Stage 2*. The residues from stage 1 above were extracted with 5 ml and of 4 M NaOH, heated to 160°C in Teflon [30]. After cooling, the mixture was filtered and later centrifuged at 5000 rpm. The supernatant was collected, and the residue was washed with deionised water. The supernatants obtained were combined and adjusted to pH ≤ 2.0 with 4 M HCL. An aliquot (5-15 ml) of the supernatant was passed through a conditioned Varian (Varian ASSOC., Harbor City, CA) Bond Elut PPL (3 ml size with 200 mg packing) solid-phase extraction tube at 5 ml/minute attached to a Visiprep (Supelco Bellefoute, PA). The tube was then placed under a vacuum (-60 kPa) until the resin was thoroughly dried after which the tube was eluted with 1 ml of ethyl acetate into gas chromatography auto sampler vials. The PPL tube was conditioned by first passing 2 ml of the ethyl acetate followed by 2 ml water (pH ≤ 2.0).

The HPLC analysis of each purified samples was then performed using Agilent 1200 series HPLC system consisting of a quaternary delivery system, an autosampler and a diode array detector (DAD). The chromatographic analysis was carried out on ChromsPher 5 column under the following conditions: using Hamilton microliter syringe (5 *μ*m × 3 mm × 250 mm) and helium gas as a carrier at a constant flow rate of 0.7 ml/minute and pressure of 180 × 10^5^ Pa. Isocratic elution mode was programmed as follows: 2% (*v*/*v*) acetic acid in water-methanol mixture 82 : 18 *v*/*v*, and UV detection was set at 320 nm. The flow rate and injection volume were set at 0.7 ml/minute and 100 *μ*l, respectively. Prior to injection, the Ackee extracts were centrifuged at 10,000 rpm for 10 minutes. The standards of different concentrations were prepared for the injection into the HPLC system for calibration and correlation coefficient establishment. The supernatants of the Ackee extracts were collected and filtrated through a 0.22 *μ*m membrane filter, and 20 *μ*l aliquots each from the filtrate were then injected into the HPLC system following the same procedure as standard mixtures.

#### 2.4.3. HPLC System (Saponin)

Prior to HPLC analysis, saponin was extracted from the samples (AL and AS). The dried methanolic extracts were pulverized, and the saponin was extracted three times with redistilled methanol. Saponin bioactive compounds were removed from 100 mg of sample using 20 ml of the redistilled methanol for 20 minutes with the aid of the sonication. The combined extract was concentrated to around 1 ml under reduced pressure and then suspended in water. The suspension was then extracted with petroleum ether, chloroform, and l-butanol saturated with water, successively, to yield the respective extract after removal of the solvent. The combined extract was filtered and concentrated to 1 ml and stored in vial bottles for further HPLC analysis.

The HPLC analysis of methanolic extracts of AL and AS was performed using HP 6890 powered with HP Chem Station Rev. A 09.01 1206 software series HPLC system consists of a flame ionization detector (FID) with 320°C detector temperature. The chromatographic analysis was carried out using capillary, DB-225MS column under the following column dimension: 30 m × 0.25 mm × 0.25 *μ*m, and oven program with initial temperature at 60°C for 5 minutes, first sampling at 12°C/minute for 18 minutes, and second sampling at 15°C/minute for 5 minutes and hydrogen pressure of 28 psi and compressed air pressure of 40 psi. Ackee extracts were centrifuged with Thermo Fisher Sorvall Legend Micro 17R, Fresco centrifuge at 10,000 rpm for 10 minutes; this was repeated 2 times. The standards of different concentrations were prepared for the injection into the HPLC system for calibration and correlation coefficient establishment. The supernatants were collected and filtered through a 0.22 *μ*m membrane filter, and 20 *μ*l aliquots from the filtrate were then injected into the HPLC system following the same procedure as standard mixtures.

### 2.5. Exposure of Cadmium Chloride to Flies and Their Survival Rate Analysis

To determine the appropriate lethal dose of CdCl_2_, 25 days survival rate analysis was carried out using 1 to 3-day-old flies. In this experiment, 1500 flies were evenly distributed into 6 groups (each group consists of five vials having 50 flies). CdCl_2_ was introduced into the diet of the flies at varying concentrations (0, 0.5, 0.75, 1.0, 1.25, and 1.5 mg/kg of diet) for each group. Daily mortality records of the flies were recorded by counting number of dead flies for 25 days and plotted as the percentage of survived flies, in order to determine their life span and survival rate, respectively, against the control (without CdCl_2_).

### 2.6. Biochemical Analysis

These experiments were designed as stated in Section 2.5; however, flies were treated only for a period of 7 days. After this incubation, the flies were collected and anesthetized with CO_2_ oozing out from a CO_2_ cylinder into the closed vials. The flies were weighed and then homogenized in 0.1 M phosphate buffer, pH 7.4 (the ratio of 1 mg: 10 *μ*l), and centrifuged at 4,000 rpm for 10 minutes at 4°C in a refrigerated centrifuge (Thermo Fisher Sorvall Legend Micro 17R, Fresco). Thereafter, supernatants were transferred into newly labelled Eppendorf tubes and used for the determination of total thiol, glutathione (GSH) contents as well as catalase, and glutathione S-transferase enzyme activities.

#### 2.6.1. Assay of Glutathione-S-Transferase Activity

The glutathione-S-transferase activity was assayed according to the method described by Ibraheem et al. using 1-chloro-2,4-dinitrobenzene (CDNB) as a substrate [33]. A total assay volume of 200 *μ*l contains 170 *μ*l of solution B (made up of 20 ml of 0.25 M potassium phosphate buffer, pH 7.0 containing 2.5 mM EDTA+10.5 ml of distilled water+0.5 ml of 0.1 M GSH), 10 *μ*l of 25 mM CDNB and 20 *μ*l of enzyme sample (1 : 5 dilution). The blank contained the same assay mixture without the enzyme. The reaction was monitored for 5 minutes at 10 seconds intervals at 25°C at 340 nm in a SpectraMax plate reader; the results were expressed as mmole/minute/mg protein using the molar extinction coefficient (*ε*) of 9.6 mM^−1^ cm^−1^ for CDNB conjugate.

(1) $$\begin{array}{c} \text{GST activity}\left(\text{mmol}/\min/\text{mg protein}\right)=\frac{{\text{Abs}}_{\left\{340\text{nm}\right\}}\times \text{reaction volume}\times \text{dilution factor}}{9.6\text{ x sample volume x mg protein}/\text{ml}}, \end{array}$$

where the extinction coefficient of CDNB/GSH = 9.6 mM^−1^ cm^−1^.

#### 2.6.2. Assay of Catalase Activity

Determination of catalase activity was carried out as described in Ibraheem et al. [33] by monitoring the clearance of H_2_O_2_ at 240 nm at 25°C [30]. A total assay volume of 590 *μ*l of solution A containing 194 *μ*l of 30% *v*/*v* H_2_O_2_ (made up to 100 ml with 50 mM potassium phosphate buffer pH 7.4) and 10 *μ*l of the enzyme sample (1 : 50 dilution) was monitored at 10 seconds intervals for 2 minutes using Jenway 6305 UV-vis spectrophotometer. The blank reaction contains the same assay mixture without the enzyme. The catalase activity was expressed as *μ*mol of H_2_O_2_ consumed/min/mg protein.

(2) $$\begin{array}{c} \text{Catalase activity }\left(\text{mmol}/\min/\text{mg protein}\right)=\frac{{\text{Abs}}_{\left\{240\text{nm}\right\}}\times \text{reaction volume}\times \text{dilution factor}}{0.0436\text{ x sample volume x mg protein}/\text{ml}}, \end{array}$$

where extinction coefficient used was 0.0436 mM^−1^ cm^−1^.

#### 2.6.3. Determination of the Concentration of Total Thiols

Total thiols level was determined following the method described by Ibraheem et al. [33]. The reaction mixture comprised 170 *μ*l of 0.1 M potassium phosphate buffer at pH 7.4, 20 *μ*l of sample and 10 *μ*l of 10 mM DTNB. The reaction mixture was incubated at room temperature for 30 minutes, after which the absorbance was measured at 412 nm with an MS033 UV-vis spectrophotometer. GSH was used as standard and expressed as mmol/mg protein, and a standard graph was plotted for each measurement.

#### 2.6.4. Assay of Hydrogen Peroxide Scavenging Activity

The H_2_O_2_ generation from lipid peroxidation was determined as described by Woff, using FOX2 reagent [34]. FOX2 reagent contained the following: 100 *μ*M Xylenol Orange, 250 *μ*M ammonium iron(II) sulfate (ferrous (NH_4_)_2_SO_4_), 90% HPLC grade methanol, 4 mM butylated hydroxytoluene (BHT), and 25 mM H_2_SO_4_. Briefly, 50 *μ*l of the test sample was added to 950 *μ*l FOX2 reagent, vortexed, and incubated for 40 minutes at room temperature and absorbance read at 580 nm with an MS033 UV-vis spectrophotometer.

#### 2.6.5. Determination of Concentration of Reduced Glutathione

This was done by the method of Jollow et al. [35]. Briefly, an equal volume of 4% *v*/*v* sulfosalicylic acid was added to the sample to deproteinize the aliquot; then, it was centrifuged using Thermo Fisher Sorvall Legend Micro 17R, Fresco centrifuge. Subsequently, 0.5 ml of the supernatant was then added to 4.5 ml of DTNB (Ellman's reagent). The absorbance at 412 nm was measured using an MS033 UV-vis spectrophotometer, and measurement was proportional to reduced glutathione.

### 2.7. Ameliorative Potentials of the AL and AS Extracts

This was done following the procedure of Ibraheem et al. who already established that 0.2 mg/g diet and 0.4 mg/g diet of the AL and AS methanolic extracts, respectively, can give the best survival and longevity when compared to the control [33]. CdCl_2_ (1.5 mg/kg of diet) was selected based on the fact that it gives the highest lethality (see Section 2.5). The ameliorative effects of the methanolic extracts of AL and AS on the CdCl_2_-induced oxidative stress were then carried out using 1 to 3-day-old flies in six groups (control, AS, AL, CdCl_2_, AL:CdCl_2_, and AS:CdCl_2_) of 50 flies in each vial for 7-day treatment (Supplementary Table 1). After, homogenization procedures as described in Section 2.6 were followed, and the biochemical assays were subsequently determined as described in Sections 2.6.1–2.6.5 were carried out.

### 2.8. Investigation of Negative Geotaxis

The negative geotaxis assay as described by Ibraheem et al. was used to determine the locomotor performance of the flies (treated and control flies) [33]. About 20 flies each in six groups (control, AS, AL, CdCl_2_, AL:CdCl_2_, and AS:CdCl_2_) were immobilized and anesthetized with CO_2_ and then placed separately in labelled vertical glass columns of length of 15 cm and diameter of 1.5 cm. 20-minute recovery from CO_2_ exposure was allowed, after which flies were gently tapped at the base of the column. The numbers of flies that climbed up to the 6 cm mark of the column in 6 seconds as well as those that remained below this mark after this time were recorded. The negative geotaxis score is expressed as the mean of the total number of flies that cross the 6 cm mark on top within the stipulated period against the total number of flies, expressed in percentage. This experiment was repeated three times at a one-minute interval.

### 2.9. Fly Emergence Rate

This assay was used to investigate the effect of CdCl_2_ and the methanolic extracts of AL and AS on the flies' emergence rate. Here, flies were treated as reported in Section 2.7, but for 24 hours. The treated diet is kept at optimum temperature needed for the flies to develop. The number of newly emerged flies is counted and compared with that of the control.

### 2.10. Protein Determination

The concentrations of protein in the various group samples (control, AS, AL, CdCl_2_, AL:CdCl_2_, and AS:CdCl_2_) were determined using 50 flies per group. The flies were weighed and then homogenized in 0.1 M phosphate buffer, pH 7.4 at a ratio of 1 mg : 10 *μ*l buffer. This was centrifuged at 4,000 rpm for 10 minutes at 4°C in a refrigerated centrifuge (Thermo Fisher Sorvall Legend Micro 17R, Fresco). Supernatants were then transferred into newly labelled Eppendorf tubes and used for the determination of the protein concentration following the Lowry method, as described in Lowry et al. [36], using bovine serum albumin (BSA) as a standard.

### 2.11. Enzyme Inhibition Studies and Determination of Kinetic Parameters (*K*_*m*_, *V*_max_, and *K*_*i*_)

#### 2.11.1. Glutathione-S-Transferase Activity

The enzyme kinetics was studied by measuring reaction rates at fixed concentration of GSH (81.35 *μ*M), varying concentrations of CDNB (49.37, 98.74, 148.11, 197.48, and 246.85 *μ*M) and at fixed concentration of CDNB (49.37 *μ*M), and varying concentrations of GSH (81.35, 162.70, 244.05, 325.39, and 406.74 *μ*M), using 10 *μ*l of enzyme (7.5 *μ*g crude protein/ml) per reaction mixture of 200 *μ*l of potassium buffer (pH 7.0). For the CdCl_2_ inhibitory effect on the enzyme, 8.18 mM of CdCl_2_ was incorporated into each reaction mixture and reaction rates measured. The apparent *K*_m_ and *V*_max_ of GST when GSH and CDNB were at varying concentrations, respectively, were measured in the absence and presence of CdCl_2_ and were determined by extrapolating them from Lineweaver-Burk plots according to the Lineweaver-Burk equation. The GSH enzyme activity was determined as previously described in Section 2.6.1.

#### 2.11.2. Catalase

The enzyme kinetics was studied by measuring the reaction rates at varying concentrations of H_2_O_2_ (0.88, 1.76, 2.65, 3.53, and 4.41 mM) using 10 *μ*l of enzyme (7.5 *μ*g crude protein/ml) per reaction mixture of 600 *μ*l of 0.1 M potassium buffer, pH 7.0. For the CdCl_2_ inhibitory effect on the enzyme, 8.18 mM of CdCl_2_ was incorporated into each reaction mixture and reaction rates were measured. The apparent *K*_m_ and *V*_max_ of catalase in the absence and presence of CdCl_2_ were determined from extrapolating them from the slope of Lineweaver-Burk plots according to the Lineweaver-Burk equation. The catalase enzyme activity was determined as previously described in Section 2.6.2.

#### 2.11.3. Determination of CdCl_2_ Inhibition Constant (*K*_*i*_)

The apparent inhibitory constant (*K*_*i*_) of CdCl_2_ for GST and catalase enzymes were obtained from Dixon plots; for GST enzyme, at a fixed concentration of CDNB (148.11 *μ*M and GSH 244.05 *μ*M), but varying concentrations of CdCl_2_ (2.73, 4.09, 5.46, 6.82, and 8.18 mM), while, for catalase enzyme, at a fixed concentration of H_2_O_2_ (2.65 mM) but varying concentrations of CdCl_2_ (2.73, 4.09, 5.45, 6.82, and 8.18 mM). The reactions were carried out following the procedures described in Sections 2.6.1 and 2.6.2, respectively.

### 2.12. Statistical Analysis

For statistical analysis, the Kaplan–Meier nonparametric method was used to analyse the survival rate and comparisons were made with the logrank test for trend. For biochemical experiments, statistical analysis was performed using a one-way analysis of variance (ANOVA) followed by Tukey's post hoc test. The results were expressed as mean ± SEM (standard error of the mean) and were considered statistically significant at *p* ≤ 0.05.

## 3. Results

### 3.1. HPLC Qualitative and Quantitative Analyses

The qualitative and quantitative identifications of the intrinsic phytochemicals present in the AL and AS methanolic extracts were identified in the HPLC chromatograms (Supplementary Figures 1A–1F), and the obtainable amounts are presented in Tables 1 and 2, respectively.

Prominent phytochemicals in the leaves are hypoglycin A, nicotine, caffeine, quinine, blighoside A, blighoside B, blighoside C, gallic acid, caffeic acid, ellagic acid, chlorogenic acid, epicatechin, myricetin, and quercetin. Also, those that are of very prominent bioactivity but are of low quantities are morphine, atropine, codeine, vanillic acid, cinnamic acid, ferulic acid, kaempferol, quercitrin, rutin, and catechin.

Prominent phytochemicals in AS are blighoside A, vanillic acid, ellagic acid, syringic acid, and gallic acid. Also, those that are of very prominent bioactivity but are of low quantities in the AS are morphine, atropine, codeine, cinnamic acid, ferulic acid, kaempferol, quercitrin, quercetin, chlorogenic acid, myricetin, caffeic acid, rutin, epicatechin, and catechin.

### 3.2. Effects of CdCl_2_ on Survival Rate of *D. melanogaster*

**Figure 1:**
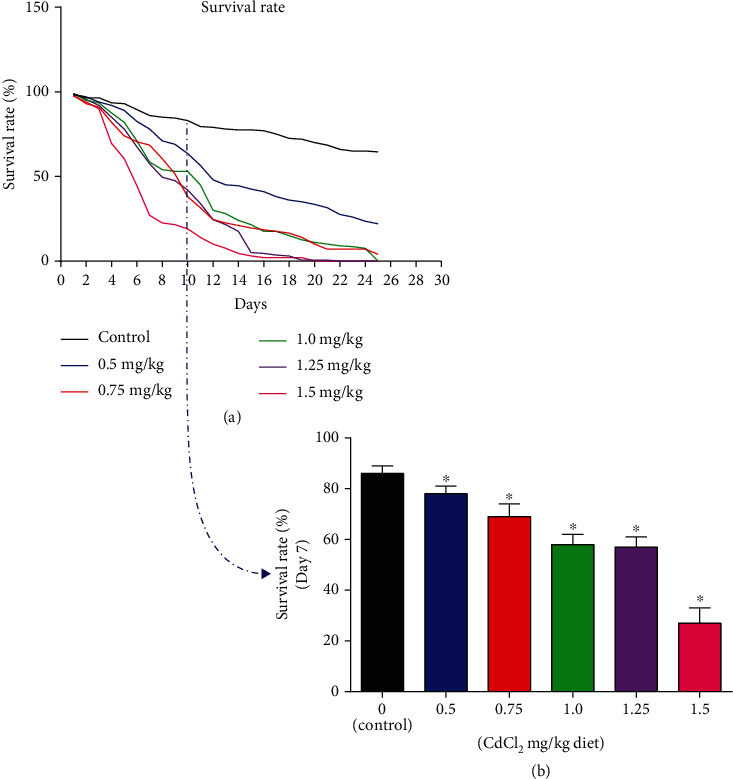
Effects of varying concentrations (0.5, 0.75, 1.0, 1.25, and 1.5 mg/Kg diet) of CdCl_2_ on the survival rate of 50 flies (both genders) of Drosophila melanogaster. (a) Survival rate after 25 days of exposure; (b) survival rate after 7 days of exposure of *D. melanogaster* to CdCl_2_. The Kaplan–Meier nonparametric method was used to analyse the survival rate and comparisons were made with the logrank test for trend. Data are presented as mean ± SEM of three independent biological replicates carried out in duplicates. ∗ indicates significant difference from control with *p* ≤ 0.05. 1.5 mg/kg diet was further studied as it gives the highest lethality at 7 days.

The effects of varying concentrations of CdCl_2_ on the survival rate of *D. melanogaster* after 25 days of exposure are shown in (Figure 1(a)). The sharp reduction in the curve shows the trends in the lethal effects of the different concentrations, depicting the increased death rate of 50 flies. 1.5 mg/kg CdCl_2_ exposed diet revealed the highest lethality when compared to control and other concentrations of CdCl_2_ exposed to the flies' diets at 7 days, and this concentration was further used for subsequent biochemical investigations (Figure 1(b)).

The effects of AL and AS methanolic extract have been reported in our previous study where 0.2 mg/g for AL and 0.4 mg/g for AS-administered *D. melanogaster* exhibited the highest survival rate compared to the control at 7 days [30].

### 3.3. Effects of CdCl_2_ Exposure on Biochemical Parameters

**Figure 2:**
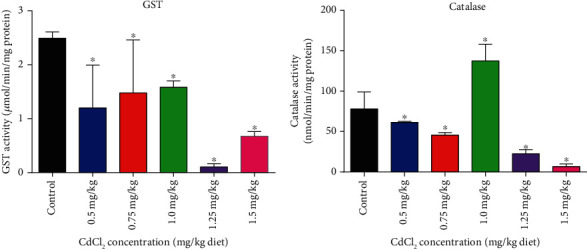
Effects of varying concentrations of CdCl_2_ on the activity of (a) GST and (b) catalase in *D. melanogaster*. Values are expressed as mean ± SEM (*n* = 5). Significant differences from the control are indicated by ∗ (*p* ≤ 0.05).

The effect of different concentrations of CdCl_2_ on GST and catalase is presented in Figure 2. The results show that GST and catalase activities of the fly groups exposed to CdCl_2_ were significantly lower when compared to control. Also, 1.25 mg/kg and 1.5 mg/kg of CdCl_2_/diet groups exhibited the lowest activities for GST and catalase, respectively. However, it was observed that the 1.0 mg/kg CdCl_2_/diet group displayed significantly higher catalase activity as compared to control.

**Figure 3:**
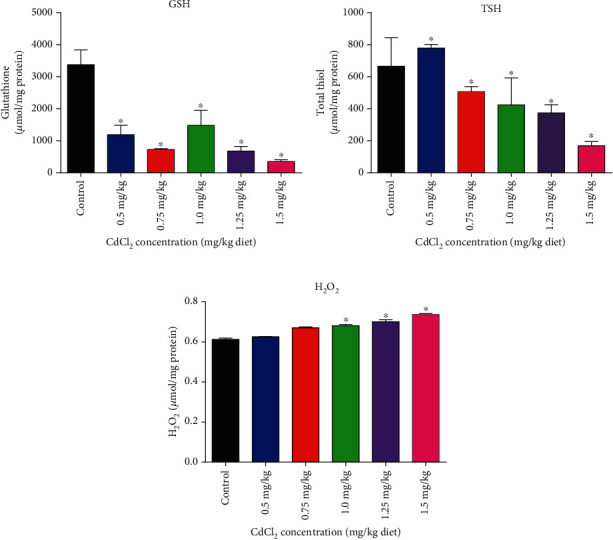
Effects of varying concentrations of CdCl_2_ on oxidative stress biomarkers in *Drosophila melanogaster*: (a) g(GSH); (b) total thiols (TSH); (c) hydrogen peroxide (H_2_O_2_). Values are expressed as mean ± SEM (*n* = 5). Significant differences from the control are indicated by ∗ (*p* < 0.05).

The effects of CdCl_2_ on the GSH, TSH, and H_2_O_2_ levels are shown in Figure 3. In the experiments, it was observed that varying concentrations of CdCl_2_ created a fluctuating pattern for the GSH and TSH levels; howbeit, 1.5 mg/kg CdCl_2_/diet still showed the lowest levels of GSH and TSH when compared to control. On the contrary, the H_2_O_2_ level increases as the concentration of CdCl_2_ increases, with the 1.5 mg/kg CdCl_2_/diet having the highest levels when compared with the control.

### 3.4. Ameliorative Potential of AL and AS Extracts

In our recent study [33], 0.2 mg/g and 0.4 mg/g methanolic extract diets of AL and AS, respectively, were able to increase the life span and survival rates of *D. melanogaster*. Thus, these concentrations were used as therapeutic doses against the CdCl_2_-induced cytotoxicity.

**Figure 4:**
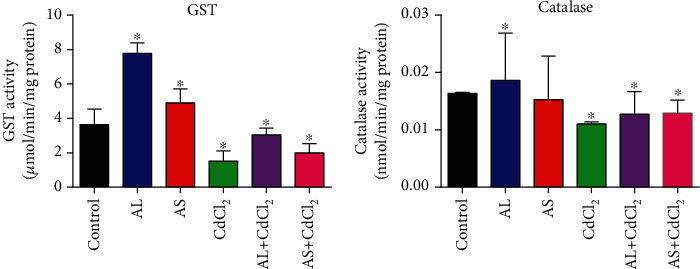
Ameliorative potentials of AL and AS on oxidative stress enzymes (a) glutathione S-transferase and (b) catalase. Values are expressed as mean ± SEM (*n* = 5). Significant differences from the control are indicated by ∗ (*p* < 0.05).

Figure 4 shows the ameliorative potentials of the AL and AS methanolic extracts towards CdCl_2_-induced toxicities on the antioxidant GST and catalase enzymes. The results showed that AL and AS methanolic extracts in the absence of CdCl_2_ increased the activity levels of GST above control, while only AL methanolic extract increased the catalase activity level above control. However, in the presence of CdCl_2_, both AL and AS methanolic extracts increased the GST and catalase enzyme level higher than the CdCl_2_-only group.

**Figure 5:**
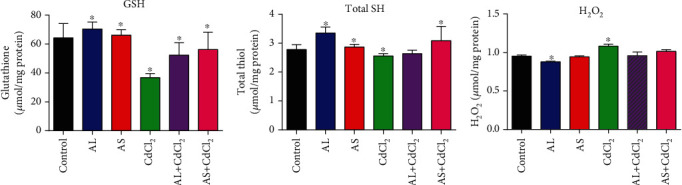
Effect of CdCl_2_ on the levels of oxidative stress biomarkers (a) glutathione (GSH), (b) total thiols (TSH), and (c) hydrogen peroxide (H_2_O_2_). Values are expressed as mean ± SEM (*n* = 5). Significant differences from the control are indicated by ∗ (*p* < 0.05).

Figure 5 likewise shows the potential of the AL and AS extracts in increasing the levels of GSH and TSH and reducing the level of H_2_O_2_ when compared to the control groups.

### 3.5. Effects of AL and AS on Negative Geotaxis and the Emergence of Offspring

**Figure 6:**
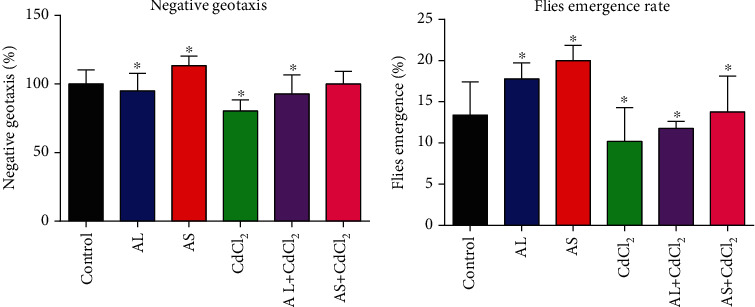
The behavioural profile of *D. melanogaster* during amelioration of CdCl_2_ toxicity by AL and AS. (a) Negative geotaxis of *D*. *melanogaster*; (b) fly emergence rates of *D. melanogaster*. Values are expressed as mean ± SEM (*n* = 5). Significant differences from the control are indicated by ∗ (*p* < 0.05).

The AL and AS methanolic extracts improved the negative geotaxis of *D. melanogaster* when compared to the control (Figure 6(a)). Furthermore, in the CdCl_2_-treated experiments, results showed that the AL and AS methanolic extracts were also able to reverse the effects of the CdCl_2_ and consequently increased the negative geotaxis with more than 10% increased activity recorded for the AL-CdCl_2_- and AS-CdCl_2_-treated groups.

Furthermore, the AL and AS extracts were able to facilitate the emergence of new offspring far higher than what was obtained in the control group (Figure 6(b)). This trend was also observed in the CdCl_2_-treated groups, where the AL-CdCl_2_ and AS-CdCl_2_ groups exhibited higher emergence rates when compared to the CdCl_2_-only group.

### 3.6. Effects of AL and AS Extracts on Protein Concentration of *D. melanogaster*

**Figure 7:**
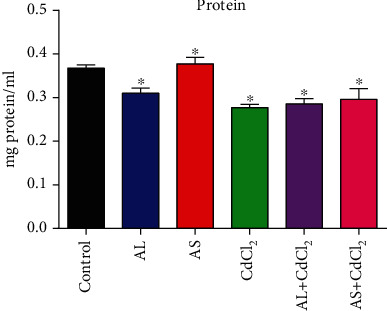
The effect of AL and AS extracts on *D. melanogaster* protein concentration during CdCl_2_ toxicity. Values were expressed as mean ± SEM (*n* = 5). Significant differences from the control are indicated by ∗ (*p* < 0.05).

Figure 7 shows the effects of the AL and AS extracts on the CdCl_2_-induced toxicities on the protein concentration. The results showed that in the absence of CdCl_2_, only aril extracts increased the protein level above control. However, in the presence of CdCl_2_, both extracts increased the protein levels higher than the CdCl_2_-only group.

### 3.7. CdCl_2_ Inhibition Kinetics on *D. melanogaster* GST and Catalase Enzymes

The CdCl_2_ inhibition studies showed that GST and catalase were inhibited by CdCl_2_ via uncompetitive and noncompetitive inhibition, respectively. Presented in Table 3 are the kinetic parameters *K*_*m*(app)_, *V*_max(app)_, and *K*_*i*(app)_ obtained from the Lineweaver-Burk plots and Dixon's plots for the GST (Supplementary Figure 2) and catalase (Supplementary Figure 3) kinetic studies. The *V*_max(app)_ for both GST and catalase enzyme activities were reduced with the introduction of the CdCl_2_ (8.18 mM).

Furthermore, the *K*_*m*(app)_ for both the GST(CDNB) and GST(GSH) activities were also reduced when compared enzyme inhibition and noninhibition assays. However, the *K*_*m*(app)_ for catalase remained the same for enzyme inhibition and noninhibition assays.

## 4. Discussion

The HPLC analysis of AL and AS phytochemicals showed that the ALs (Table 1) have higher yields of alkaloid, saponin, and phenolic contents when compared to AS (Table 2). However, the AS methanolic extract possesses higher contents of phenolic compound. Gallic acid, vanillic acid, ferulic acid, syringic acid, caffeic acid, isoquercitrin, ellagic acid, rutin, neochlorogenin, and hecogenin. Many research works have demonstrated numerous biological and pharmacological potentials these plant-derived biochemicals could confer such as antidiabetics, antiulcer, antitumor and anti-inflammatory, antiatherogenic, antiaging, antiallergic, antithrombotic, and antimutagenic properties [37].

Our study revealed that AL and AS methanolic extracts possess appreciable amount of quercetin and catechins. It has been reported that quercetin has great biological properties such as anticarcinogenic, antiobesity, and antiproliferative activities, inhibits adipogenesis, and induces cell death [38].

Furthermore, some fruits containing flavonoids such as catechins have been found to lower coronary mortality and protect from complicated diseases like hepatic disorder, cancer, and neurodegenerative diseases [39]. It has been shown that green tea's antioxidant activity is mainly due to caffeine, catechin, epicatechin, and other polyphenols and black tea's antioxidant activity is attributed to quercetin, while coffee's antioxidant activity is attributed to chlorogenic acid [40]. Thus, regular tea intake has been established to be involved in the reduction of risks associated with neurodegenerative diseases such as Alzheimer's disease, Parkinson disease, dementia, stroke, and even coronary heart disease [41]. Also, quercetin therapy has been employed in renal ischemia/reperfusion increased GSH levels, enhancing the antioxidant system in rat models. [42]. Quercetin treatment was reported to have reduced the level of malondialdehyde and increased the level of superoxide dismutase and catalase after lipopolysaccharide induction, thus suggesting the enhancement of oxidative stress defense system by quercetin in a rat experimental model [43]. Therefore, the presence of appreciable amounts of these noble bioactive compounds in AL and AS shows the beneficial potential that Ackee plant parts could confer towards their usage as therapeutic agents for treatment of different human ailments. Flavonoids have been shown to have an important role in preserving biological systems or macromolecules such as proteins, carbohydrates, lipids, and DNA against free radical oxidation [44].

Furthermore, chlorogenic acid is found in fruits and herbs such as tomatoes, apples, carrots, and coffee beans, and studies have shown that they possess antioxidant and anti-inflammatory [45], antidiabetic, and antilipidemic properties [46]. Chlorogenic acid is used for the prevention and treatment of metabolic syndrome and associated disorders such as type 2 diabetes mellitus and cardiovascular diseases [47]. The prominent level of these compounds in AL reveals the probable therapeutic actions of these AL in the management of illnesses and diseases. Also, the content of ellagic acid in the methanolic extracts of AL (44.06 mg/100 g) and AS (114.42 mg/100 g) is higher compared to those in black raspberry (38.00 mg/100 g), blackberry (43.67 mg/100 g) [48], strawberry (1.24 mg/100 g) [49], cloudberry (15.30 mg/100 g), and red raspberry (2.12 mg/100 g) [50]. Several experimental evidence has shown that ellagic acid has promising potential against diverse forms of cancer cells, such as osteocarcinoma, gliobastoma, oral cancer, ovarian cancer, and hepatocarcinoma [51]. Thus, the extracts of Ackee plant parts could be highly harnessed towards the therapeutic treatment of different illnesses.

Fruit such as Açaí palm fruit (*Euterpe oleracea*) has been shown to have abundant presence of syringic acid which has been reported to have good therapeutic effects in bone resorption or osteoporosis in the ovariectomized mouse models [52]. It was reported that low dosages of caffeic acid and syringic acid could protect neurons from ischemic insults [53], reduce blood pressure, and prevent organ damage in hypertensive rats [54]. Interestingly, our HPLC analysis showed very high levels of syringic acid in the AS methanolic extract. This therefore highlights the efficacy of AS as a valuable resource towards the management of these diseases. Extracts from plants such as Ceylon cinnamon (*Cinnamomum verum*) [55], Jaboticaba (*Plinia cauliflora*) [56], Limonium (*Nelumbo nucifera*) [57], and *Ocimum basilicum* [58] have been reported to be rich in gallic acid and have all displayed ameliorative potentials against obesity-related diseases. Regular consumptions of fruits such as avocado [59] and blackcurrant [60] which are rich in gallic acid were shown to be associated with health benefits against obesity related diseases. In this study, gallic acid was prominent in both AL and AS; however, it was 5 times higher in the AS extract. Thus, AL and AS will be valuable resources in the treatment and/or management of obesity related diseases.

Myricetin is typically an integral component in the epicarps of many fruits such as red grapes, and it has also been found at high levels in vegetables, green tea, and wine [61]. It has been reported to have higher free radical scavenging activity when compared to some other flavonoids, and it inhibits lipid peroxidation [58]. Studies have shown that myricetin exerts its anticarcinogenic effect by inhibiting enzymes that activate carcinogens, modifying signal transduction pathways, interacting with other cancer associated proteins [62], and inhibiting angiogenesis [63]. The prominent presence of myricetin in AL methanolic extract may thus support the traditional use of AL in cancer therapy and/or management.

Vanillic acid is also commonly found in edible plants, fruits, green tea, and coffee [64]. It possesses antioxidant, antihypertensive, and hepatoprotective properties in addition to protecting against cardiac toxicity [65] and snake venom-induced toxicity [66]. Studies have shown that rutin ameliorates the oxidative effect of mercuric chloride (HgCl_2_) on catalase, GSH, and other antioxidant defense system in rat liver by increasing these antioxidant enzyme levels, also in methotrexate-induced hepatotoxicity in rat model [67]. Nafees et al. also showed various pharmacological activities against allergy, bacterial, ulcer, oxidative stress, carcinogens, and diabetes, among others [68]. Our study thus revealed the medicinal potentials that AS which are very rich in both vanillic acid and rutin could confer towards the therapeutic management of these different diseases.

The AL extract contains slightly higher amount of hypoglycin A and B than the AS extract (Tables 1 and 2). This was due to the fact that the Ackee fruit from where the AS were harvested was allowed to reach maturity, allowing the Ackee fruit pods to open before harvesting. This allowed the reduction in the quantities of hypoglycin A and B in AS as compared to the leaves. Hypoglycin is linked to acute toxic effects, hypoglycemia syndrome, and even death in consumption of unripened AS thus limiting their use in some communities [69]. Blighosides A, B, and C have been isolated from the pods of the Ackee fruit [70]. These classes of blighosides have all been shown to have an antiproliferative impact on human breast cancer cells [71]. Caffeine, nicotine, and morphine all have stimulating properties, and they have been employed as analgesics, while quinine has been utilized as a component of antimalarial drugs to treat malaria disease [61]. Apigenin, kaempferol, and luteolin have been shown to have inverse association with coronary heart disease mortality [61]. It was revealed that in dogs and monkeys, they were efficient inhibitors of platelet aggregation [72] and they are also potent antiviral agents because of the nonglycosidic chemicals in them and their hydroxylation at the 3rd position [73]. Furthermore, apigenin from peas or garlic, epicatechin from green tea, and quercetin from the outer skin of onions were shown to possess several protective effects against radiation-induced damage [74]. In rats, kaempferol was shown to exhibit antiulcerogenic properties [75], while catechins, tannins, coumarins, and other plant-derived compounds were shown to interfere with HIV reverse transcriptase, integrase, and protease [76]. Taken together, the diverse phytochemicals inherent in AL and AS are highly relevant in their traditional application towards the treatment and/or management of various illnesses.

Cadmium is a biohazardous environmental toxic metal that has been shown in this study to shorten the lifespan of *D. melanogaster* at concentrations of 0.5, 0.75, 1.0, 1.25, and 1.5 mg/kg. In our previous study, AL and AS offered a diversity of activities, including lowering H_2_O_2_ levels and increasing GST, GSH, and TSH levels in flies given AL and AS diets. Giustarini et al. in their work documented that during CdCl_2_-induced oxidative stress in flies, oxidation of GSH cysteine residues can result in the reversible formation of mixed disulfides between protein thiol groups, GSH, and other low-molecular-mass thiols (S-thiolation), thus lowering the total thiol content or GSH/GSSG in the flies biological system temporarily until antioxidative enzymes are activated to reverse the effect [77]. This correlates with reports from Provan et al. where the independent administration of HgCl_2_ impaired antioxidant defense system, thereby increasing hydrogen peroxide stress, and decreased glutathione, total thiol level, catalase, and GST activities in *D. melanogaster* system [30].

Furthermore, cadmium has been shown to reduce catalase expression in the Wistar rat [78] which correlates with results obtained in this study of catalase activity of cadmium-exposed *D. melanogaster*. A report has also shown that GSH and some other chelators in plants play a vital role in the detoxification of cadmium [79, 80]. It was also shown that cadmium activates changes in antioxidant defense systems, resulting in the reduction of GSH and increase in glutathione-S-transferase and catalase activities in rats [80]. This correlates with results for GST and catalase activities in *D. melanogaster* system in this study (Figures 2 and 3). Also, it has been reported that catalase activity was enhanced in *T. pisana* exposed to Pb^2+^ and Cu^2+^ [81] and in Cu, Zn, and Pb pollutant-exposed *C. aspersus* [82]. It has been shown that the exposure of several terrestrial land snails and gastropod species to metals such as Pb^2+^ and Cd^2+^ enhanced their GST activity level [83], and this adjustment to toxicant-induced stress includes both detoxifying actions and antioxidant defense [84]. It has also been reported that cadmium binding to the SH group in the mitochondria and secondary injury initiated by the activation of Kupffer cells are the likely mechanism for the toxicity of Cd^2+^ in the hepatocytes of rat liver [85].

In this study, AL-fed flies had higher catalase activity when compared to the CdCl_2_-only fed flies, demonstrating the antioxidant potential of AL in reducing the oxidative effects of CdCl_2_. When compared to the control value, the higher level of GSH in AL, higher level in AL+CdCl_2_, and lower level in CdCl_2_-only diets suggest the antioxidant potential of AL (Figures 4 and 5). The AL, AS, and control diet fed flies had a greater TSH level or GSH/GSSG ratio, indicating a substantially increased state of total thiol content in the *D. melanogaster* cellular environment, because GSH was abundant as cysteine storage before the CdCl_2_-induced oxidative environment. This correlates with our previous study where the main phytochemicals in AL and AS offered a diversity of activities, including lowering H_2_O_2_ levels and increasing GST, GSH, and TSH levels in flies given AL and AS diets [33].

In the untreated *D. melanogaster*, the control, AL, and AS-fed flies had lower H_2_O_2_ levels (Figure 5(c)); however, of the three, AL had the highest catalase activity. According to prior studies, the biological response to H_2_O_2_ should have increased catalase activity as H_2_O_2_ load increases in the cadmium-induced oxidative stress, in order to break down the extra H_2_O_2_ into water and oxygen molecules [85]; however, catalase activity decreased (Figure 4(b)). It is therefore imperative to study the mechanism of Cd^2+^ inhibition on catalase enzyme which this study has established. In our previous study, increased catalase activity brought about reduction in H_2_O_2_ concentration in HgCl_2_-induced oxidative stress [33]. Also, in another study where flies were treated with Al^3+^, it was shown that there was an increased catalase activity after two days [86]. Nevertheless, after a longer period, catalase activity is reduced and this reduction was attributed to an increased in the release of free radicals, thereby exerting inhibitory effects on catalase activity [87]. This clearly indicates that each heavy metal has its unique way of inhibiting catalase enzyme or any other oxidative stress-associated enzymes.

Report has shown that the administration of 200 mg/kg *Xylocarpus granatum* bark extract to oxidative stress-induced diabetic mice increased catalase and superoxide dismutase activities, while glutathione peroxidase reduced to a normal level, thus depicting an increased antioxidant defense in response to the induction of diabetes in rat liver [88]. This is in accordance with this study where AL and AS methanolic extracts increased GST and catalase activities in *D. melanogaster* (Figure 4), whereas there was a decline in GST and catalase activities as a result of CdCl_2_-induced oxidative stress. Contrary to this is the report of Koutsogiannaki et al. [89] and Cong et al. [90] who claimed that Cd^2+^ can increase antioxidant gene expression during oxidative stress and that Cd^2+^ can trigger the enhancement of GST activity as a compensatory mechanism to increase oxidative stress in Wistar rats. A study showed an enhancement of the myocardium catalase activity after administering ethanol in rats at a chronic level [91]. Cd^2+^ reduction of catalase and GST activity in our study is in accordance with Fouad and Jresat [92] and Wang et al. [93] who suggested that the reduction in GST and catalase activities by cadmium was due to the formation of transition complex between cadmium and the protein enzymes, thus inhibiting the activities of these enzymes and their synthesis at transcription level, thereby increasing production of reactive oxygen species [83].

A fly's negative geotaxis profile refers to how quickly it can climb vertically after being tapped to the bottom of a vessel as part of its natural escape response. Negative geotactic ability has been shown to be susceptible to oxidative stress [94], and our result (Figure 6(a)) shows an activity pattern similar to GST, catalase, and TSH results (Figures 4 and 5). Adjusting one or two commonly used ingredients in fly meals, or adding toxicants to be examined to the diets, has become a standard procedure in order to establish the impact of the alterations on reproductive rates and longevity, both of which are essential markers [20]. The emergence rate of CdCl_2_-only fed flies was lower as compared to the control value, whereas the emergence rate of AL and AS extracts was higher than the control value, thus demonstrating the influence of the Ackee extracts on reproductive profile and their therapeutic value on the emergence rate of the flies when exposed to toxicants such as Cd^2+^ [19] (Figure 6(b)). This correlates with studies where *Catharanthus roseus* extracts reduced the toxic effects of endosulfan and its isomers, scavenged the free radicals produced by them, and protected *D. melanogaster* from oxidative damages [95]. Also, the coadministration of *C. roseus* extracts along with toxic endosulfan and its isomers has been shown to improve fecundity, fertility, and reproductive performance of the *D. melanogaster* [96], which is in relation with the ameliorative roles of AL and AS on fly negative geotaxis and emergence rate in our study (Figure 6).

Report have shown that Ag^+^ is a more potent inhibitor after comparing its inhibitory effects to Cu^2+^, Mg^2+^, Fe^2+^, Zn^2+^, Ni^2+^, and others on purified GST from turkey liver [97]. It was also revealed that Ag^+^ was the best inhibitor having studied the inhibitory effects of certain metallic ions on the activity of purified GST enzyme from *C. tarichii* (Pallas muscle tissue) [98]. Comakli et al. having compared the inhibitory effects of Mg^2+^, Cd^2+^, Cr^2+^, and Ag^+^ on GST on rainbow trout erythrocytes reported that Ag^+^ had higher inhibition effects when compared to others [99]. The inhibition effects of Cd^2+^ and Cu^2+^ on these erythrocytes were noncompetitive, and this was not the same with the uncompetitive inhibition of Cd^2+^ with GST of *D. melanogaster* in this study (Supplementary Figure 2), whereas Ag^+^ and Zn^2+^ were competitive while Pb^2+^, Fe^3+^, and Cr^2+^ had no inhibitory activity on GST [100]. Uncompetitive inhibition is commonly observed in chemical reactions involving more than one substrate or products [101]. Cd^2+^ binds to the [GST–GSH] complex forming [GST-GSH-Cd^2+^] complex, thereby preventing the other substrates from binding and transforming into products. The [GST-GSH-Cd^2+^] complex will continue to form, and invariably, there will be no product transformation.

If the [GST-GSH] complex reduces, why was there a decrease in *K*_*m*_? The affinity should have increased and not reduced. The fact is that for the inhibitor to be active, the [GST-GSH-Cd^2+^] complex must be tightly bound and the GST enzyme binds tighter to the GSH after binding to Cd^2+^ making the inhibition effective, since Cd^2+^ is not easily displaced by another substrate, hence the reduction in *K*_*m*_, i.e., higher affinity binding (Supplementary Figure 2A; Table 3). Furthermore, the [GST-CDNB-Cd^2+^] complex also exhibited uncompetitive inhibition. The Cd^2+^ binds to the [GST–CDNB] complex forming the [GST-CDNB-Cd^2+^] complex, thus preventing the other substrate from binding and transforming into product. Similarly, for the Cd^2+^ to be active, the [GST-CDNB-Cd^2+^] complex must be tightly bound, the GST binds tighter to CDNB after binding to Cd^2+^ making the inhibition effective, and Cd^2+^ cannot be easily displaced by another substrate, hence the reduction in *K*_*m*_, higher affinity binding (Supplementary Figure 2B; Table 3). In the presence of Cd^2+^, enzyme activity will not be higher even as GSH and CDNB concentrations are increased. But at low concentration of GSH and CDNB, the difference in enzyme activity will be minimal (Table 3).

On the other hand, the Cd^2+^ inhibition constant *K*_*i*(app)_ obtained for GST enzyme (2.13 mM) was far higher than the *K*_*m*_ for the respective substrates (GSH, 1.29 mM; CDNB, 0.49 mM). These values were further reduced on introduction of the Cd^2+^ (GSH, 0.39 mM; CDNB, 0.29 mM), an indication that in the presence of an inhibitor the enzyme, GST bound to the substrate is quite higher and the ability to relieve itself from the inhibition is reduced. Furthermore, increase in the TSH concentration as observed in Figure 5(b) gave stronger indication of the ability of the system to overcome the Cd^2+^ inhibitory effect. Consequently, GST has higher affinity for the Cd^2+^ in the presence of GSH and CDNB than it has with GSH or CDNB alone.

The [catalase-H_2_O_2_-Cd^2+^] complex could either be transformed to the [catalase-Cd^2+^-H_2_O_2_] or the [catalase–Cd^2+^] complex in the presence of both H_2_O_2_ and cadmium been bonded to the catalase, thus preventing the [catalase–Cd^2+^] complex from completing a chemical reaction. This has no effect on catalase's *K*_*m*_ (affinity) for H_2_O_2_, thus indicating the noncompetitive inhibition (Supplementary Figure 3A). However, the drug deferasirox (Fe(III) chelator) competitively inhibited catalase, thereby making the binding of the deferasirox to the catalase enzyme to prevent the binding of the hydrogen peroxide substrate and vice-versa [102]. It was reported that cyanide and azide are potent ligands that can bind to the haem group of catalase [103] and hence inhibit enzyme activity. Azide inhibitory effect on catalase activity has been considered a rapid and reversible inhibition, in the presence of oxygen and high H_2_O_2_/azide ratio [104].

This inhibition action of azide on catalase is different from the inhibitory effects of Cd^2+^ on catalase, as cadmium may bind to an allosteric site, and this may have occurred in a direct reversible or irreversible manner. From this study, cadmium may have bound to an allosteric location of catalase other than the H_2_O_2_ binding site, forming both [catalase-Cd^2+^] and [catalase-Cd^2+^-H_2_O_2_] complexes and inhibiting the [catalase-H_2_O_2_] complex process. The removal of cadmium reduces catalase turnover without affecting the amount of H_2_O_2_ that binds to the enzyme. Thus, there is observably no change in *K*_*m*_-binding affinity to H_2_O_2_. Furthermore, the Cd^2+^ inhibition constant [*K*_*i* (app)_] obtained for catalase enzyme (1.72 mM) was apparently very close to the enzyme *K*_*m*_ (1.09 mM) an indication that increase in the substrate concentration may not relieve the inhibitory effect imposed by Cd^2+^ on the enzyme and hence the observable significant decrease in catalase activity (Figure 4(b)) despite the increasing H_2_O_2_ concentration (Figure 5(c)).

This also brought about the noncompetitive inhibitory pattern of Cd^2+^ on catalase that has very similar affinity for both Cd^2+^ and H_2_O_2_. The inhibition constant (*K*_*i*{app}_) of Cd^2+^ on catalase enzyme is higher when compared with the *K*_*m*_ of catalase on H_2_O_2_ when Cd^2+^ is bound. This shows that catalase has lower affinity for the Cd^2+^ in the presence of hydrogen peroxide; thus, the catalytic efficiency of catalase may be enhanced and prevent the [catalase-Cd^2+^] complex from been formed, which may either involve blocking the allosteric site where Cd^2+^ binds or introducing another ligand into the system that will specifically bind to Cd^2+^, consequently preventing it from binding to the catalase enzyme.

This study has therefore established the mechanism of action of the Cd^2+^ enzyme inhibition. It is believed that the Cd^2+^inhibits both GST and catalase by binding to the enzyme catalytic active or substrate binding site or the inhibition may be involved in the change in the catalytic active site conformation, which may prevent substrate binding, consequently leading to an upsurge in the substrate concentration. These phenomena could have been all that ensued in this study as evident in the ameliorative roles of AL and AS methanolic extracts that reverse/relieve the enzymes of the Cd^2+^ inhibition. Thus, the AL and AS phytochemicals must be acting as a scavenger of the CdCl_2_, thereby reversing Cd^2+^ inhibitory effects on GST and catalase enzymes and allowing them to convert the deleterious compounds (CDNB and H_2_O_2_) to less harmful compounds that could be easily eliminated.

## 5. Conclusion

The existence of bioactive chemicals in AL and AS has played significant roles in their antioxidant activities and the validity of the tradomedical claims among some ethnic groups. The HPLC analyses identified various alkaloid, phenols, and saponins in the methanolic extracts of AL and AS by HPLC analysis. When compared to the control at *p* ≤ 0.05, AL and AS extracts showed remarkable ameliorative activity on Cd^2+^-induced oxidative stress on GST and catalase activities by increasing their activity levels and GSH level and reducing the H_2_O_2_ production level in *D. melanogaster*. The Ackee plant parts may have participated in binding to the Cd^2+^, thus preventing them from binding to the enzymes catalytic sites, or they could have participated in removing or breaking down the Cd^2+^ inhibition complex, thereby relieving the enzyme of the Cd^2+^ inhibitory consequences, facilitating the release and enablement of the enzymes (GST and catalase) back to their native state, and also seek to the tighter binding of the Cd^2+^-phytochemical complexes which eventually will lead to the Cd^2+^ detoxification and elimination by appropriate cognate organs.

It will be of very great interest if this phenomenon could be further studied structurally and functionally and also to identify and establish which of these phytochemicals inherent in the AL and AS methanolic extracts actually participated in the ameliorative activity. This information will be very vital and may be exploited in future research towards the design of pharmacological agents that could confer similar ameliorative roles not only to Cd^2+^-induced cytotoxicity in humans, but also to other known toxicants such as Cu^2+^, Ag^+^, Ni^2+^, Cr^2+^, and Al^3+^. Taken together all our findings, AL and AS could be beneficial in the treatment/management of heavy metal-associated cytotoxicity or oxidative stress in humans.

## Figures and Tables

**Table 1:** Different bioactive components and their quantities expressed as mg/100 g sample of AL methanolic extract.

Alkaloid	Amount (mg/100 g sample)	Phenolics	Amount (mg/100 g sample)	Saponin	Amount (mg/100 g sample)
Hypoglycin A	1.2403	Phenol	0.0141	Tigogenin	0.0772
Hypoglycin B	0.3787	Vanillic acid	0.0242	Neochlorogenin	0.0282
Nicotine	1.4871	P-Hydroxybenzoic acid	0.0216	Hecogenin	0.7609
Coniine	0.0040	Cinnamic acid	0.1043	Blighoside A	114.13
Morphine	0.0163	Protocatechuic acids	0.1774		0.0544
Lupanine	0.0387	P-coumaric acid	0.3310	Blighoside B	3.7606
Caffeine	3.3286	O-coumaric acid	0.0344	Blighoside C	3.5193
Galantamine	0.0006	Gallic acid	17.6366		
Atropine	6.58 × 10^−4^	Caffeic acid	79.4551		
Quinine	2.0722	Ferulic acid	0.1391		
Thebaine	0.0768	Syringic acid	0.3218		
Cinchonine	0.0827	Ellagic acid	44.0564		
Codeine	1.19 × 10^−3^	Sinapinic acid	0.4677		
Senecionine	2.623 × 10^−3^	Chlorogenic acid	165.4319		
Quinidine	0.0506	Quercetin	96.9039		
Berberine	2.88 × 10^−3^	Kaempferol	0.6510		
Magnoflorine	1.15 × 10^−3^	Quercitrin	0.0358		
Sparteine	0.0202	Isoquercitrin	0.0161		
Solanidine	0.0491	Rutin	0.1251		
Cinchonidine	0.1051	Apigenin	0.4137		
Colchicine	0.0644	Hesperidin	0.0101		
Aconite	0.3586	Catechin	0.4135		
		Epicatechin	27.4270		
		Myricetin	33.4333		
		Naringenin	0.1762		

**Table 2:** Different bioactive components and their quantities expressed as mg/100 g sample of AS methanolic extract.

Alkaloid	Amount (mg/100 g sample)	Phenolics	Amount (mg/100 g sample)	Saponin	Amount (mg/100 g sample)
Hypoglycin A	0.0434	Phenol	0.0172	Tigogenin	0.04690
Hypoglycin B	0.0454	Vanillic acid	178.8601	Neochlorogenin	0.0673
Nicotine	0.1887	P-hydroxybenzoic acid	0.0070	Hecogenin	0.8462
Coniine	0.00075	Cinnamic acid	0.0312	Blighoside A	65.4996
Morphine	0.00167	Protocatechuic acids	0.0529	Tribuloin	0.0389
Lupanine	0.0079	P-coumaric acid	0.0822	Blighoside B	1.2190
Caffeine	0.5790	O-coumaric acid	0.0195	Blighoside C	2.6226
Galantamine	1.1 × 10^−4^	Gallic acid	89.2598		
Atropine	9.08 × 10^−4^	Caffeic acid	1.1267		
Quinine	0.1854	Ferulic acid	0.3345		
Thebaine	0.0129	Syringic acid	47.6779		
Cinchonine	0.0235	Ellagic acid	114.4161		
Codeine	1.32 × 10^−4^	Sinapinic acid	0.2435		
Senecionine	2.43 × 10^−4^	Chlorogenic acid	0.1583		
Quinidine	0.0177	Quercetin	1.7837		
Berberine	4.18 × 10^−4^	Kaempferol	0.2580		
Magnoflorine	6.11× 10^−4^	Quercitrin	0.0105		
Sparteine	0.0119	Isoquercitrin	0.0198		
Solanidine	0.0080	Rutin	0.1913		
Cinchonidine	0.0412	Apigenin	0.1807		
Colchicine	0.0558	Hesperidin	0.0036		
Aconite	0.1743	Catechin	0.2363		
		Epicatechin	0.2606		
		Myricetin	0.3875		
		Naringenin	0.1389		

**Table:** (a) Kinetic parameters obtained for GST and catalase in the absence or presence of CdCl_2_

Enzyme	*V*_max{app}_ (−Inh)mmolmin^−1^ mg^−1^protein	*V*_max{app}_ (+Inh)mmolmin^−1^ mg^−1^protein	*K*_*m*{app}_ (−Inh; mM)	*K*_*m*{app}_ (+Inh; mM)	*V*_max{app}_ (−Inh)/*K*_m(app)_ (−Inh)	*V*_max{app}_ (+Inh)/*K*_m(app)_ (+Inh)
GST[CDNB]	5.79	3.47	0.49	0.29	11.91	11.97
GST{GSH}	6.72	2.15	1.29	0.39	5.61	5.45
Catalase [H_2_O_2_]	10314.22	7754.59	1.09	1.09	9500.06	7210.22

**Table:** (b) Kinetic parameters obtained from Dixon plots

Inhibition	*V*_max{app}_ (+Inh) mmolemin^−1^ mg^−1^protein	*K*_*i*_ (mM)	*V*_max(app)_(+Inh)/*K*_*i*_ (mM)
Cd^2+^_(GST)_	20.8	2.13	9.77
Cd^2+^_(Catalase)_	16383.03	1.72	9508.43

## Data Availability

All experimental data obtained from this study and used in this publication are available upon request from the corresponding author.

## Abbreviations

AL: Ackee Leaves
AS: Ackee Arils
Cd^2+^: Cadmium Ion
CdCl_2_: Cadmium Chloride
H_2_O_2_: Hydrogen Peroxide
GSH: Reduced Glutathione
GST: Glutathione S-Transferase
ROS: Reactive Oxygen Species
RNS: Reactive Nitrogen Species.

## Supplementary Materials

Supplementary 1 Supplementary Table 1 illustrates the feeding procedures employed in order to evaluate the ameliorative potentials of the AL and AS extracts on the CdCl_2_-induced oxidative stress in the 1-3 days old *D. melanogaster.*

Supplementary 2 Supplementary Figure 1A–1F shows the obtained HPLC chromatograms for the qualitative and quantitative identification of phytochemicals present in the Ackee (*Blighia sapida* K.D. Koenig) leaves and aril methanolic extracts used in this study. The identified phytochemicals and the obtainable amounts are presented in Tables 1 and 2 for AL and AS, respectively.
