# Supplementary material for: Ackee (Blighia sapida K.D. Koenig) Leaves and Arils Methanolic Extracts Ameliorate CdCl2-Induced Oxidative Stress Biomarkers in Drosophila melanogaster
Source: Oxid Med Cell Longev. 2022 Nov 14;2022:3235031. doi: 10.1155/2022/3235031 (PMC9679428; doi:10.1155/2022/3235031)
Supplement: Supplementary 1 — Supplementary Table 1 illustrates the feeding procedures employed in order to evaluate the ameliorative potentials of the AL and AS extracts on the CdCl2-induced oxidative stress in the 1-3 days old D. melanogaster. [file 3235031.f1.docx]

**Supplementary Table 1: Different treatments exposure on the 50 flies in each vial for 7 days**

| GROUP | Treatment | Concentration |
| --- | --- | --- |
| 1 | Control | 0 |
| II | Arils (AS) | 0.4 mg/g diet |
| III | Ackee leaves (AL) | 0.2 mg/g diet |
| IV | CdCl_2_ | 1.5 mg/Kg diet |
| V | Ackee leaves (AL) : CdCl_2_ | 0.2 mg/g diet : 1.5 mg/Kg diet |
| VI | Arils (AS) : CdCl_2_ | 0.4 mg/g diet : 1.5 mg/Kg diet |

Table illustrates the various treatments in order to evaluate the ameliorative effects of these methanolic extracts of Ackee leaves and arils on the CdCl_2_ induced oxidative stress carried out on 1-3 days old flies.
