# Supplementary material for: Ackee (Blighia sapida K.D. Koenig) Leaves and Arils Methanolic Extracts Ameliorate CdCl2-Induced Oxidative Stress Biomarkers in Drosophila melanogaster
Source: Oxid Med Cell Longev. 2022 Nov 14;2022:3235031. doi: 10.1155/2022/3235031 (PMC9679428; doi:10.1155/2022/3235031)
Supplement: Supplementary 2 — Supplementary Figure 1A–1F shows the obtained HPLC chromatograms for the qualitative and quantitative identification of phytochemicals present in the Ackee (Blighia sapida K.D. Koenig) leaves and aril methanolic extracts used in this study. The identified phytochemicals and the obtainable amounts are presented in Tables 1 and 2 for AL and AS, respectively. [file 3235031.f2.docx]

Figure 1a: HPLC Chromatographic analysis of Ackee leaves different alkaloids constituents

Figure 1b: HPLC Chromatographic analysis of Ackee leaves different phenolic constituents

Figure 1c: HPLC Chromatographic analysis of Ackee leaves different saponins constituents

Figure 1d: HPLC Chromatographic analysis of Ackee arils different alkaloids constituents

Figure 1e: HPLC Chromatographic analysis of Ackee arils different phenolic constituents

Figure 1f: HPLC Chromatographic analysis of Ackee arils different **s**aponins constituents

Figure 2: The Cd^2+^ inhibitory effects on GST enzyme in *D.melanogaster*. Where: (A) the Lineweaver-Burk plot of GST at varying concentration of GSH, while CDNB concentration is fixed (49.37 µM); (B) the Lineweaver-Burk plot of GST at varying concentrations of CDNB, while GSH concentration is fixed (81.35 µM); (C) the Dixon plot for determination of the Cd^2+^ inhibition constant (K_i_) at fixed CDNB (148.11 µM) and GSH (244.05 µM), varying the concentrations of CdCl_2_.

Figure 3: The Cd^2+^-inhibitory effect on catalase enzyme in *D.melanogaster*. Where: (A) the Lineweaver-Burk plot of catalase at varying concentrations of H_2_O_2_; (B) the Dixon plot for determination of the Cd^2+^ inhibition constant (K_i_) at fixed H_2_O_2_ [2.65 mM], varying the concentrations of CdCl_2_.
